# Coregulation of *FANCA* and *BRCA1* in human cells

**DOI:** 10.1186/2193-1801-3-381

**Published:** 2014-07-28

**Authors:** Anneke Haitjema, Berber M Mol, Irsan E Kooi, Maarten PG Massink, Jens AL Jørgensen, Davy AP Rockx, Martin A Rooimans, Johan P de Winter, Hanne Meijers-Heijboer, Hans Joenje, Josephine C Dorsman

**Affiliations:** Department of Clinical Genetics, Section Oncogenetics, VU University Medical Center, Van der Boechorststraat 7, 1081 BT Amsterdam, The Netherlands

**Keywords:** Fanconi anemia, Functional genomics, Retinoblastoma, Breast cancer, FANCA, BRCA1

## Abstract

Fanconi anemia (FA) is a genetically heterogeneous syndrome associated with increased cancer predisposition. The underlying genes govern the FA pathway which functions to protect the genome during the S-phase of the cell cycle. While upregulation of FA genes has been linked to chemotherapy resistance, little is known about their regulation in response to proliferative stimuli. The purpose of this study was to examine how FA genes are regulated, especially in relation to the cell cycle, in order to reveal their possible participation in biochemical networks. Expression of 14 FA genes was monitored in two human cell-cycle models and in two RB1/E2F pathway-associated primary cancers, retinoblastoma and basal breast cancer. *In silico* studies were performed to further evaluate coregulation and identify connected networks and diseases. Only *FANCA* was consistently induced over 2-fold; *FANCF* failed to exhibit any regulatory fluctuations. Two tools exploiting public data sets indicated coregulation of *FANCA* with *BRCA1*. Upregulation of *FANCA* and *BRCA1* correlated with upregulation of *E2F3*. Genes coregulated with both *FANCA* and *BRCA1* were enriched for MeSH-Term id(s) genomic instability, microcephaly, and Bloom syndrome, and enriched for the cellular component centrosome. The regulation of FA genes appears highly divergent. In RB1-linked tumors, upregulation of FA network genes was associated with reduced expression of *FANCF*. FANCA and BRCA1 may jointly act in a subnetwork - supporting vital function(s) at the subcellular level (centrosome) as well as at the level of embryonic development (mechanisms controlling head circumference).

**Electronic supplementary material:**

The online version of this article (doi:10.1186/2193-1801-3-381) contains supplementary material, which is available to authorized users.

## 1 Background

Fanconi anemia (FA) is a rare, recessive, genetically heterogeneous, chromosomal instability disorder, characterized by developmental abnormalities, retarded growth, bone marrow failure, and a high risk for the development of cancer (Auerbach et al. 2001; Alter 2003; Rosenberg et al. 2003; Kutler et al. 2003). Fanconi anemia patient-derived cells are extremely sensitive to bifunctional alkylating or DNA interstrand cross-linking agents, such as mitomycin C and cisplatin (Ishida and Buchwald 1982; Wang 2007).

Currently, sixteen FA genes have been identified, each corresponding to a distinct ‘complementation group’: FA-A, -B, -C, -D1, -D2, -E, -F, -G, -I, -J, -L, -M, -N, -O, -P, and -Q (Strathdee et al. 1992; Pronk et al. 1995; Apostolou et al. 1996; Lo Ten Foe et al. 1996; de Winter et al. 1998, 2000a, b; Waisfisz et al. 1999; Timmers et al. 2001; Howlett et al. 2002; Meetei et al. 2003, 2004, 2005; Levitus et al. 2005; Levran et al. 2005a; Dorsman et al. 2007; Xia et al. 2007; Smogorzewska et al. 2007; Sims et al. 2007; Vaz et al. 2010; Kim et al. 2011; Stoepker et al. 2011; Bogliolo et al. 2013); the most common groups being FA-A, -C, and -G, together accounting for 85% of all FA patients (Levran et al. 2005b; de Winter and Joenje 2009).

To maintain genome integrity, the FA proteins function together in the so-called FA/BRCA-pathway to repair DNA damage, such as double strands breaks (DSBs). The FA/BRCA-pathway is divided into an upstream and a downstream branch in relation to the monoubiquitination of FANCD2 and FANCI, which is considered a central activating reaction. This reaction is catalyzed by the so-called core complex, which is thought to be assembled via subcomplexes. These complexes are FANC-A and -G; FANC-B and -L; FANC-E, -C, and -F. Together with FANCM these proteins constitute the core complex (Medhurst et al. 2006). The activation of FANCD2 and FANCI coordinates the activities of FA proteins that act downstream in the pathway leading to DNA repair: FANCD1/BRCA2, FANCJ/BRIP1, FANCN/PALB2, FANCO/RAD51C, FANCP/SLX4, FANCQ/ERCC4/XPF and XRCC2. Homozygous germ line mutations in *BRCA1* can result in a Fanconi anemia-like phenotype (Domchek et al. 2013; D’Andrea 2013), *BRCA1* may thus be considered an FA-like gene whose action may thus be closely connected to the FA pathway.

In addition to their involvement in the canonical FA/BRCA-pathway acting either in the upstream, central, or downstream part, additional protein complexes of FA proteins have been described that may serve distinct or related functions, such as FANCD1/BRCA2-FANCD2-FANCG-XRCC3 (Wilson et al. 2008), and FANCA-BRCA1 (Folias et al. 2002). Moreover, some FA proteins appear to function in additional, seemingly unrelated, processes such as oxidative metabolism, cell cycle progression, apoptosis, and transcriptional regulation, which may be relevant for some of the pathological features of FA (Kaddar and Carreau 2012).

Biallelic mutations in the genes underlying the FA/BRCA-pathway cause predisposition to malignancies in FA patients. On the other hand, there is evidence that a proportion of cancers arising in the (non-FA) general populations (‘sporadic cancers’) may possess a disrupted FA/BRCA pathway (Stecklein and Jensen 2012). The status of this pathway appears to be relevant for cancer treatment response. Repression has been associated with a favorable response against cross-linking drugs (Chen et al. 2005; Stecklein and Jensen 2012), whereas hyperactivation might be responsible for resistance to such drugs.

Upregulation of several FA genes, especially during S phase, has been linked to the RB1/E2F pathway (Tategu et al. 2007; Hoskins et al. 2008; Kim and D’Andrea 2012), which is known to control cell cycle progression (Nevins 2001; Chen et al. 2009; Knudsen and Wang 2010). This pathway plays an important role in transcriptional regulation during the cell cycle. Proteins of the RB1 family, pRb, p107 and p130 work together with the sequence-specific DNA-binding factors of the E2F family which consists of the activators E2F1-E2F3 and repressors E2F4-E2F8 (Chen et al. 2009; Di Fiore et al. 2013). During growth arrest, E2F activity can be repressed by the RB1 protein family via protein-protein interactions, while during progression to the cell cycle from G1 to S phase phosphorylation of the RB1 family members results into E2F activation (Henley and Dick 2012). Since disruption of the RB1/E2F pathway and upregulation of E2F target genes is frequently observed in human cancers (Nevins 2001; Chen et al. 2009; Knudsen and Wang 2010) upregulation of (a subset) of FA genes may be a common feature for tumors with a disrupted RB1 pathway.

In this study we monitored expression of 14 FA genes during the cell cycle and in cancers with a disrupted RB1/E2F pathway, in an attempt to identify gene expression patterns that characterize two important interconnected pathways, i.e. the FA and RB1/E2F pathways.

## 2 Results

### 2.1 Upregulation of the FA mRNA level upon progression through the cell cycle in two cell models

**Figure 1:**
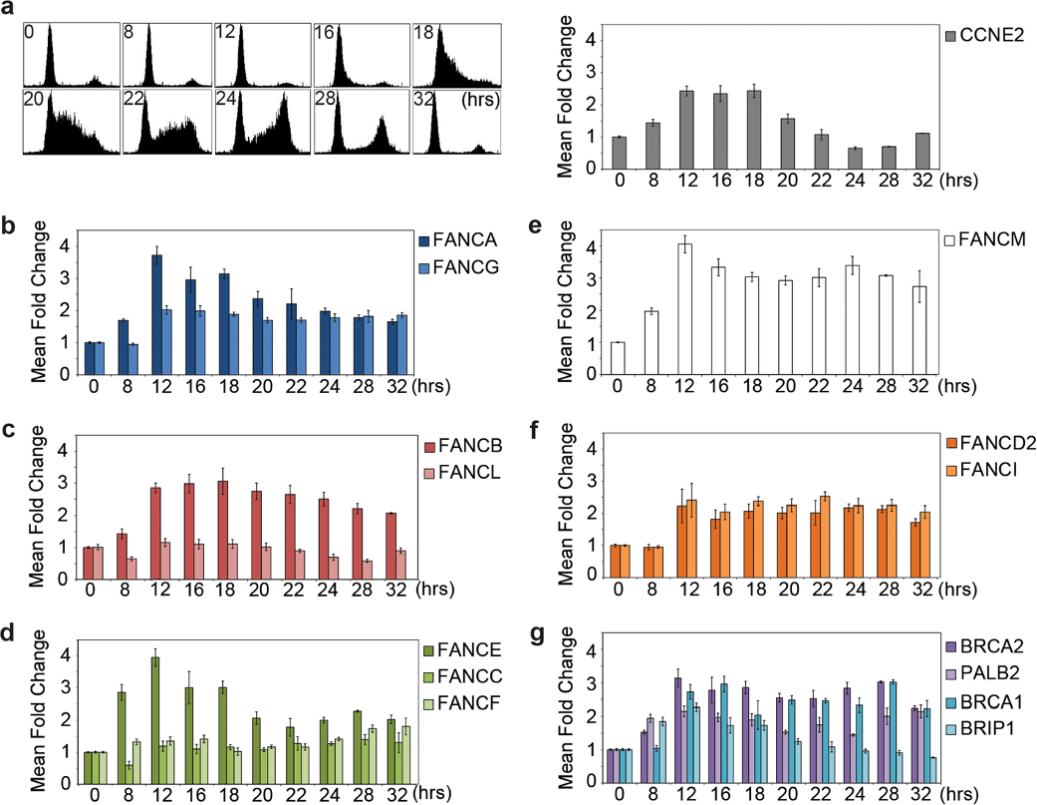
**Differential cell cycle regulation of FA genes in human T98G cells. (a-left panel)** FACS analysis of synchronized cells, the 0, 8, 12, 16, 18, 20, 22, 24, 28, and 32 hr time points are shown; S phase is most pronounced between 18–22 hrs. Data of FACS represents one representative synchronization **(a-right panel)** Control experiment: The mRNA levels of *CCNE2* during the cell cycle are shown. Quantitative RT-PCR was performed on total RNA samples from different time points and mean fold changes (MFC) were calculated relative to time point zero. Data represents the average mean fold change of 2–3 independent synchronization each in duplicate qPCR measurement (except for time points: 28 and 32) **(b-g)** Relative gene expression during the cell cycle for genes encoding in the FA/BRCA-pathway.

To study the expression of FA genes during the cell cycle, we used the established human cell-cycle model T98G derived from glioblastoma cells (Stein 1979). T98G cells can be efficiently arrested via serum deprivation, while after serum stimulation a synchronized progression through the cell cycle can be observed. T98G cells express abundant levels of E2F activity and possess a functional RB pathway (Takahashi et al. 2000). Fluorescent Activated Cell Sorting (FACS) analysis and *CCNE2* expression confirmed the proper synchronization of the cells (Figure 1a - left and right panel).

The mRNA expression levels of the following endogenous FA genes (*FANC-A, -B, -C, -E, -F, -G, -L, -M*, *-D2*, *-I*, *-D1*, *-N*, *-J*, and *BRCA1*) were analyzed with RT-qPCR. Changes in mRNA levels were subsequently calculated relative to time point zero. Several of the FA genes turned out to be prominently induced during the cell cycle progression (Figure 1b-g), e.g. *FANCA* (Figure 1b) while other FA genes, like *FANCL* (Figure 1c) and *FANCF* (Figure 1d) showed a relatively constitutive expression pattern during the cell cycle.

We also compared the expression of endogenous FA genes during the cell cycle in human diploid fibroblasts (EVA-F) via serum deprivation (Additional file 1: Figure S1). FACS analysis (Additional file 1: Figure S1a - left panel) and expression analysis of *CCNE2* confirmed the proper synchronization of the cells (Additional file 1: Figure S1a - right panel). In this instance, also a subset of the FA genes turned out to be prominently upregulated during the cell cycle relative to time point zero, again *FANCA* (Additional file 1: Figure S1b), but also *FANCD2* (Additional file 1: Figure S1f) and *BRIP1* (Additional file 1: Figure S1g); while other FA genes, like again *FANCL* (Additional file 1: Figure S1c) and *FANCF* (Additional file 1: Figure S1d), were less affected during the cell cycle progression.

### 2.2 Upregulation of FA gene expression in cancers associated with disrupted RB1/E2F pathway

To study the upregulation of the FA/BRCA-pathway in cancers with a disrupted RB1/E2F pathway, two different primary human tumors, retinoblastoma (Rb) and basal breast cancer were studied. The vast majority of retinoblastomas harbors mutations in *RB1* (Dunn et al. 1988), while it has been recently recognized that functional loss of *RB1* is a common event in basal like breast tumors (Herschkowitz et al. 2008). To discriminate between upregulation caused by altered regulation of expression or by DNA copy number alteration (CNA), we determined the correlation between CNA and mRNA expression of FA/BRCA genes (Henrichsen et al. 2009; Dear 2009; Kuiper et al. 2010).

#### 2.2.1 Upregulation of FA genes in *RB1-*mutated retinoblastoma versus fetal retina

We analyzed the gene expression in a cohort of primary retinoblastoma tumors and compared that to healthy fetal retina samples. We found a significant upregulation in retinoblastoma tumors (most significant Fold Changes [FCs] indicated, for all probes and corresponding FCs see Table 1), for the core complex members: *FANCA* (FC = 3.54, P = 2.32E-06), *FANCC* (FC = 1.48, P = 3.25E-03), *FANCE* (FC = 1.93, P = 2.07E-02), *FANCG* (FC = 4.00, P = 1.52E-07), *FANCL* (FC = 2.65, P = 4.95E-09), *FANCM* (FC = 1.51, P = 2.08E-02); central players: *FANCD2* (FC = 1.95, P = 4.68E-03) and *FANCI* (FC = 2.69, P = 1.55E-03); and for the downstream branch: *FANCD1/BRCA2* (FC = 2.90, P = 4.65E-04), *FANCN/PALB2* (FC = 2.18, P = 8.70E-09), and *BRCA1* (FC = 2.05, P = 2.13E-04) compared to healthy fetal retina. For the core complex member *FANCF* we found a significant downregulation (FC = -1.81, P = 1.30E-04). For the core complex member *FANCB* and downstream branch member *FANCJ/BRIP1* no significant differences were detected compared to healthy fetal retina. The upregulation of *FANCE* was driven by CNA since a linear statistical significant correlation was found between CNA and mRNA expression (FDR P-value cut-off 5.00E-02; Table 2).

**Table 1:** **Gene expression in Retinoblastoma tumors versus fetal retina**

Part	Gene symbol	Probe ID	Fold change	P-value^*^
Core complex	FANCA	203805_PM_s_at	2.20	6.06E-03
	FANCA	203806_PM_s_at	3.54	2.32E-06
	FANCA	236976_PM_at	4.00	2.68E-06
	FANCC	242654_PM_at	1.48	3.25E-03
	FANCC	205189_PM_s_at	1.49	2.77E-02
	FANCC	1559513_PM_a_at	1.52	6.28E-03
	FANCE	220255_PM_at	1.93	2.07E-02
	FANCF	218689_PM_at	-1.81	1.30E-04
	FANCF	222713_PM_s_at	-1.37	2.65E-02
	FANCG	203564_PM_at	4.00	1.52E-07
	FANCL	218397_PM_at	2.65	4.95E-09
	FANCM	234733_PM_s_at	1.51	2.08E-02
	FANCM	242711_PM_x_at	1.56	2.27E-02
Central players	FANCD2	242560_PM_at	1.95	4.68E-03
	FANCI	223785_PM_at	1.69	2.47E-02
	FANCI	213008_PM_at	2.55	3.08E-03
	FANCI	213007_PM_at	2.69	1.55E-03
Downstrem branch	BRCA2	214727_PM_at	2.31	5.87E-03
	BRCA2	208368_PM_s_at	2.90	4.65E-04
	PALB2	219530_PM_at	2.18	8.70E-09
	BRCA1	204531_PM_s_at	2.05	2.13E-04
Acitvating E2Fs	E2F1	204947_PM_at	1.61	5.47E-03
	E2F1	2028_PM_s_at	1.86	2.56E-03
	E2F2	228361_PM_at	2.84	2.31E-04
	E2F3	203692_PM_s_at	3.50	1.94E-08
	E2F3	203693_PM_s_at	3.72	2.18E-10
Control	CCNE2	211814_PM_s_at	7.77	3.13E-10
	CCNE2	205034_PM_at	8.88	1.22E-11

^*^Cutoff P-value < 0.05.

**Table 2:** **Significant correlation copy number variation and mRNA in retinoblastoma**

Gene symbol	Probe ID	Linear correlation	P-value	FDR P-value^*^
FANCE	220255_PM_at	2.25	2.03E-07	3.24E-05
E2F3	203692_PM_s_at	1.65	1.99E-08	5.78E-06
E2F3	203693_PM_s_at	1.50	1.81E-08	5.34E-06

^*^Cutoff FDR P-value < 0.05.

Interestingly, in *MYCN*-amplified (without *RB1* mutations) retinoblastoma (Rushlow et al. 2013) compared to *RB1-*mutated retinoblastomas, we found a significant downregulation of the core complex members: *FANCA* (FC = -3.99, P = 3.69E-03), *FANCC* (FC = -1.91, P = 1.46E-02), *FANCL* (FC = -2.56, P = 2.72E-06), *FANCM* (FC = -2.07, P = 2.16E-02); central player: *FANCI* (FC = -3.74, P = 1.56E-02); and downstream branch member *BRCA1* (FC = -2.36, P = 8.85E-03; Additional file 2: Table S1) compared to classic retinoblastoma (with *RB1* mutations). This provided additional evidence that the disrupted RB1/E2F-pathway in the *RB1-*mutated retinoblastoma tumors may play a role in upregulation of the FA/BRCA-pathway members.

#### 2.2.2 Upregulation of FA genes in basal versus not-basal breast tumors

We compared 41 basal breast tumors (27 *BRCA1*-mutated) and 79 not-basal breast tumors (8 *BRCA1* mutated) for FA gene expression (Table 3). A significant upregulation in basal breast tumors compared to not-basal tumors was found for the core complex members: *FANCA* (FC = 3.40, P = 1.93E-13), *FANCB* (FC = 4.73, P = 5.18E-12), *FANCC* (FC = 1.38, P = 3.38E-04), *FANCE* (FC = 1.62, P = 2.80E-08), *FANCG* (FC = 1.39, P = 1.52E-06), *FANCL* (FC = 1.52, P = 3.79E-06); central players: *FANCD2* (FC = 1.66, P = 4.57E-06) and *FANCI* (FC = 1.98, P = 1.46E-06); and for the downstream branch: *FANCD1/BRCA2* (FC = 1.96, P = 3.45E-09), and *FANCJ/BRIP1* (FC = 1.96, P = 5.75E-05). A significant downregulation was found for the core complex members *FANCF* (FC = -1.29, P = 2.60E-03), and *FANCM* (FC = -1.28, P = 1.26E-02). No significant differences were detected for the downstream branch member: *FANCN/PALB2*. The expression status of *BRCA1* is influenced by mutation status therefore we consider it unknown.

**Table 3:** **Gene expression in basal breast tumors versus not-basal**

Part	Gene symbol	Probe ID	Fold change	P-value^*^
Core complex	FANCA	215530_at	1.51	6.41E-03
	FANCA	236976_at	2.07	7.57E-04
	FANCA	203806_s_at	3.06	2.74E-12
	FANCA	203805_s_at	3.40	1.93E-13
	FANCB	243597_at	1.95	5.10E-05
	FANCB	1553244_at	2.38	1.26E-05
	FANCB	1557217_a_at	3.06	3.02E-07
	FANCB	1557218_s_at	4.73	5.18E-12
	FANCC	205189_s_at	1.38	3.38E-04
	FANCE	220255_at	1.62	2.80E-08
	FANCF	218689_at	-1.29	2.60E-03
	FANCG	203564_at	1.39	1.52E-06
	FANCL	218397_at	1.52	3.79E-06
	FANCM	242711_x_at	-1.28	1.26E-02
Central players	FANCD2	242560_at	1.66	4.57E-06
	FANCD2	223545_at	1.74	2.22E-05
	FANCI	223785_at	1.60	1.17E-05
	FANCI	213007_at	1.80	1.53E-06
	FANCI	213008_at	1.98	1.46E-06
Downstrem branch	BRCA2	208368_s_at	1.96	3.45E-09
	BRCA2	214727_at	1.76	6.74E-08
	BRIP1	221703_at	2.04	1.28E-03
	BRIP1	221703_at	2.04	1.28E-03
	BRIP1	221703_at	2.04	1.28E-03
	BRIP1	235609_at	1.96	5.75E-05
Acitvating E2Fs	E2F1	2028_s_at	1.63	1.89E-05
	E2F1	204947_at	2.01	8.72E-06
	E2F2	235582_at	1.55	2.28E-02
	E2F2	228361_at	1.98	2.97E-07
	E2F3	203692_s_at	1.95	8.13E-17
	E2F3	203693_s_at	2.21	1.12E-12

^*^Cutoff P-value < 0.05.

The correlation between CNA and mRNA expression of FA/BRCA genes was determined in the basal breast tumors (n = 41; Table 4). Statistical significant linear correlation (FDR P-value cut-off 5.00E-02) was found for *FANCI* and *FANCF* showing a strong linear correlation (> 0.60), suggesting that differences in gene expression are driven by CNAs.

**Table 4:** **Significant correlation copy number variation and mRNA in basal-like tumors**

Gene symbol	Probe ID	Linear correlation ^*^	P-value	FDR P-value^**^
FANCI	213007_at	0.77	3.23E-09	1.61E-07
FANCF	222713_s_at	0.73	4.93E-08	1.23E-06
FANCI	213008_at	0.72	9.63E-08	1.61E-06
E2F3	203693_s_at	0.70	4.05E-07	5.06E-06
FANCF	218689_at	0.68	1.19E-06	1.19E-05
E2F3	203693_s_at	0.66	3.04E-06	2.54E-05
E2F3	203692_s_at	0.60	3.75E-05	2.68E-04

^*^Cutoff > 0.60; ^**^Cutoff FDR P-value < 0.05.

### 2.3 *E2F*involvement

To determine which E2F transcription factors could be associated with upregulation of FA genes, we studied also the mRNA expression levels of activating E2F genes (*E2F1*, *E2F2*, *E2F3*) in *RB1-*mutated retinoblastoma and in basal breast tumors.

In *RB1-*mutated retinoblastoma, all three activating E2Fs were significantly upregulated: *E2F1* (FC = 1.86, P = 2.56E-03), *E2F2* (FC = 2.84, P = 2.31E-04), and *E2F3* (FC = 3.72, P = 2.18E-10; Table 1). Upregulation of the activating E2F genes was also found in basal versus not-basal breast cancer: *E2F1* (FC = 2.01, P = 8.72E-06), *E2F2* (FC = 1.98, P = 2.97E-07), and *E2F3* (FC = 1.95, P = 8.13E-17; Table 3). Importantly, *E2F3* upregulation was in both cases (retinoblastoma and basal breast cancer) driven by a CNA, since a strong correlation was found between CNA and mRNA expression (FDR P-value cut-off 5.00E-02; Tables 2 and 4).

### 2.4 Differential regulation of FA/BRCA genes

**Figure 2:**
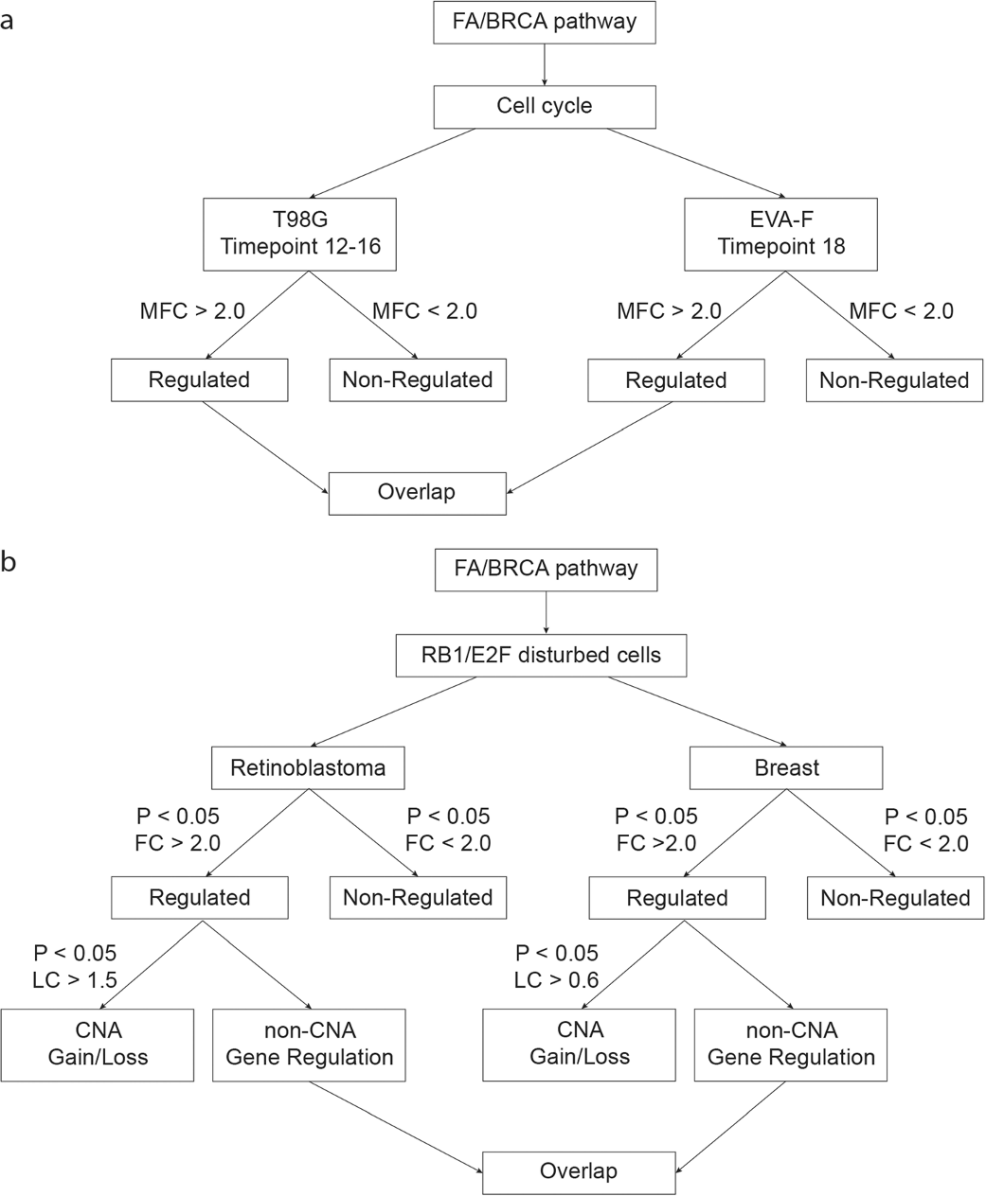
**Decision tree(s) to select coregulated genes. (a)** Decision tree for selecting FA/BRCA genes that are regulated through the cell cycle. **(b)** Decision tree to select FA/BRCA genes in RB1/E2F disturbed cells. Two cell models: retinoblastoma and breast cancer cells are screened. In both models a selection is made for “Regulated” or “Non-Regulated”. In case of “Regulated” a selection is made on the possible influence of copy number alterations (CNA). Abbreviations: P = P-value; MFC = mean fold change; FC = fold change; LC = linear correlation; CNA = copy number alteration.

Established cell-cycle and E2F target genes, such as *CCNE2* are commonly robustly regulated by proliferative stimuli (see also Figure 1 and Additional file 1: Figure S1). To further group FA/BRCA genes in relation to cell cycle regulation and induction by proliferative stimuli we used two decision trees (Figure 2). For each criterion in the decision trees, genes received points which are summarized in Table 5.

**Table 5:** **Scoring scheme regulated expression FA/BRCA genes**

		Cell cycle model		RB1/E2F disturbed cancers		
Part	Gene symbol	T98G	EVA-F	Retinoblastoma	Basal breast tumors	Total
Core complex	FANCA	1	1	1	1	4
	FANCB	1	1	0	1	3
	FANCC	0	1	0	0	1
	FANCE	1	0	0	0	1
	FANCF	0	0	0	0	0
	FANCG	1	1	1	0	3
	FANCL	0	1	1	0	2
	FANCM	1	1	0	0	2
Central players	FANCD2	1	1	0	0	2
	FANCI	1	1	1	0	3
Downstream branch	FANCD1/BRCA2	1	1	1	0	3
	FANCN/PALB2	1	1	1	0	3
	BRCA1	1	1	1	0	3
	FANCJ/BRIP1	1	1	1	0	3

1 point = regulated; 0 = not regulated.

In the first approach, cell cycle regulated genes were selected based on their behaviour in the human cell models. In the T98G model the average value of time point 12–16 should have a MFC > 2.0, while in the diploid fibroblasts time point 18 should have a MFC > 2.0 (Figure 2a). This approach allows to interrogate induction prior to entry of S phase; the usual pattern for regulation at the mRNA level for genes playing a role in S phase. Based on these criteria, “regulated” genes turned out to be the core complex members: *FANCA*, *FANCB*, *FANCG*, and *FANCM*; central players: *FANCD2* and *FANCI*; and for the downstream branch: *FANCD1/BRCA2*, *FANCN/PALB2*, *FANCJ/BRIP1*, and *BRCA1* (Additional file 2: Table S2). The core complex member *FANCE* was only considered regulated in the T98G cell line, whereas *FANCC* and *FANCL* only in the EVA-F fibroblasts. The core complex member *FANCF* was in both cell types considered not regulated.

In the second approach, to select for proliferation stimulated genes, we used the criterion ‘fold change > 2’ in combination with significant P-values (Figure 2b) based on their behaviour in RB1-linked cancers. Upregulation of FA genes was found in both the retinoblastoma as well as in basal breast tumors. Only a few expression differences were possibly driven by CNA (*FANCE*, *FANCF*, and *FANCI*). The only gene that was consistently upregulated in both retinoblastoma as well as in basal breast tumors was *FANCA* (Additional file 2: Table S3).

The results of the combined scoring (Figure 2) are summarized in Table 5; a gene can have maximal 4 points, reflecting induction in all 4 contexts. Only *FANCA* turned out to have 4 points. The following genes obtained all 3 points: *FANCB*, *FANCG*, *FANCI*, *FANCD1/BRCA2*, *FANCN/PALB2*, *BRCA1*, and *FANCJ/BRIP1. FANCF* turned out not to be upregulated in both cell models and in the primary tumors its expression was also low which was correlated with CNA. The results indicate differential regulation of FA genes in response to proliferative stimuli, with *FANCA* the most regulated.

#### 2.4.1 *In silico*analysis to determine coregulation of FA/BRCA genes

To further study coregulation, we performed *in silico* studies using the most regulated gene, i.e. *FANCA*, as the starting point. Firstly, we analyzed the correlation of *FANCA* with other genes with help of two online annotation tools, BioGPS (Wu et al. 2009) and GeneFriends (http://genefriends.org/microArray/) (van Dam et al. 2012). With the first tool BioGPS the dataset GeneAtlas U133A, gcrma was analyzed (Su et al. 2004). This allowed us to interrogate the overall gene expression profile of a panel of 79 different human tissues, including several cell lines from the NCI-60 cancer cell panel. The analysis of the gene expression pattern of *FANCA* indicated a strong correlation (> 0.7) with *BRCA1* (0.7851), and *FANCE* (0.7402; Table 6).

**Table 6:** **Gene correlation mRNA expression pattern with** ***FANCA***

Symbol	Reporter	Correlation^*^
**FANCA**	**203806_S_AT**	**1.0000**
CCDC85A	GNF1H07976_AT	0.9214
SUSD3	GNF1H08030_AT	0.8978
NLRP11	GNF1H07113_AT	0.8752
RPAIN	216962_AT	0.8719
WDR43	214662_AT	0.8525
PNPT1	GNF1H09065_S_AT	0.8510
AICDA	219841_AT	0.8462
C3orf37	201678_S_AT	0.8370
PRAMEF24P	GNF1H08246_AT	0.8104
E2F5	221586_S_AT	0.8014
BACH2	221234_S_AT	0.7903
RFC1	208021_S_AT	0.7869
MRPL48	GNF1H02267_S_AT	0.7864
	217464_AT	0.7853
**BRCA1**	**204531_S_AT**	**0.7851**
BFSP2	207399_AT	0.7657
RMI2	GNF1H08947_AT	0.7636
TCF3	209152_S_AT	0.7603
LRMP	204674_AT	0.7549
GCSAM	GNF1H07830_AT	0.7536
	GNF1H03417_S_AT	0.7525
ELL3	219518_S_AT	0.7523
SHMT2	214095_AT	0.7442
**FANCE**	**220255_AT**	**0.7402**
ZNF804A	215767_AT	0.7352
ALDH5A1	203608_AT	0.7301
ZNF232	219123_AT	0.7244
LRMP	35974_AT	0.7173
MTHFD1L	GNF1H02482_S_AT	0.7126
ISG15	205483_S_AT	0.7115
PRDM15	GNF1H10126_AT	0.7032

^*^Correlation Cutoff: > 0.7; Bold: Genes of interest.

The second tool GeneFriends identifies co-expressed genes in a genome wide co-expression map over 4,000 human microarray datasets. The underlying datasets were derived from a variety of conditions. Searching GeneFriends with *FANCA* returns in the top list *FANCA* itself with a co-expressed value of 1.0 as expected. Intriguingly, only co-expression of *FANCA* with *BRCA1* was found and not with other FA/BRCA genes in the top 50 list. The co-expression value of *FANCA* with *BRCA1* was 0.708 indicating that *FANCA* is increased in expression (≥2 fold) in 70.8% of the cases when *BRCA1* is increased in expression (≥2 fold; Additional file 2: Table S4). The strong correlation of *FANCA* with *BRCA1* found with two different approaches, suggest that these two genes are frequently co-expressed. With GeneFriends, we further analyzed which genes are frequently coregulated with both *FANCA* and *BRCA1* (Additional file 2: Table S5). Enrichment analysis of this gene set revealed Medical Subject Heading Terms (MeSH; top 3; Additional file 2: Table S6) genomic instability (P-value: 4.42E-04), microcephaly (P-value: 8.33E-04), and Bloom syndrome (P-value: 3.33E-03) and an enrichment for the cellular component centrosome (GO:0005813; P-value: 3.55E-08; Additional file 2: Table S7).

#### 2.4.2 Coregulation of *FANCA*and *BRCA1*

**Figure 3:**
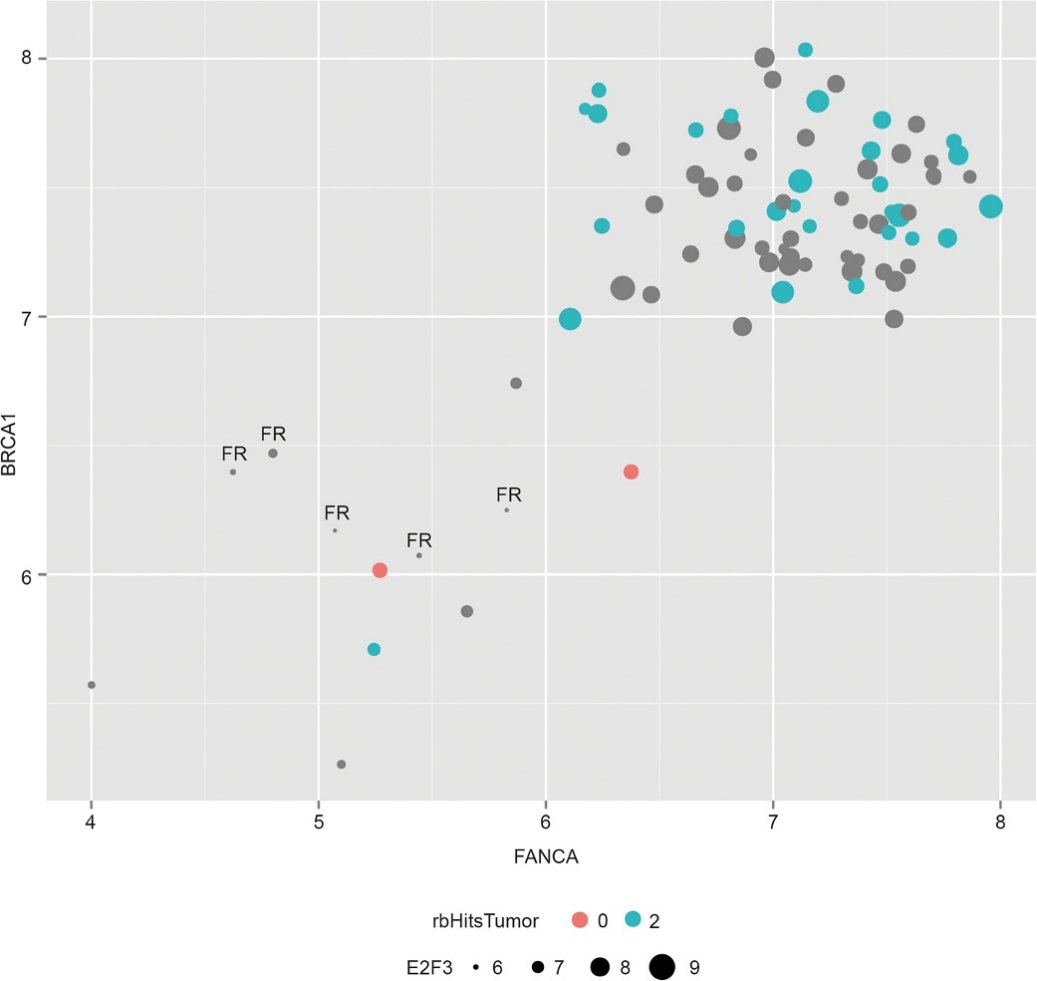
**FANCA and BRCA1 are coexpressed in Retinoblastoma tumors.** Correlation of normalized intensities of the most significant probe sets of *FANCA* (x-axis: 203806_PM_s_at, P = 2.32E-06) and *BRCA1* (y-axis: 204531_PM_s_at, P = 2.13E-04) demonstrates a strong correlation (Pearson = 0.72). Retinoblastoma with no *RB1* mutations but with high *MYCN* amplification (orange/red dots) have relatively low expression of *FANCA* and *BRCA1*. Dot sizes indicate levels of *E2F3* expression. Retinoblastoma linked to *RB1*; blue dots: *RB1* mutation determined in tumor; grey dots: *RB1* mutation not determined in tumor. FR = Fetal Retina.

Combining the results of the studies in the cell models and the tumors with the *in silico* data (Figure 2; Table 5; and Additional file 2: Table S4), reveals a high degree of coregulation of *FANCA* and *BRCA1*, with *E2F3* as a possible important driver for cell-cycle regulated expression. To evaluate the degree of *FANCA*-*BRCA1* co-expression, we measured the correlation coefficient of *FANCA* and *BRCA1* expression in the Rb-tumor cohort (Figure 3). A strong linear correlation was found for co-expression of *FANCA* and *BRCA1* (Pearson: 0.72). Interestingly, when adding the *E2F3* expression to the *FANCA* and *BRCA1* gene expression correlation, tumor samples with relatively low expression of *FANCA* and *BRCA1* had also low *E2F3* and vice versa (Figure 3). This suggests a possible role for *E2F3* as a key driver for the gene regulation of the studied genes, and in particularly *FANCA* and *BRCA1*. Three established E2F3 target genes, *CCNE1*, *FEN1*, and *PCNA*, displayed a similar pattern (results not shown). As noted above, the Rb samples with *MYCN* amplifications have relative low level of expression for both genes.

## 3 Discussion and conclusion

We found evidence for highly divergent cell cycle regulation of FA pathway genes, with *FANCA* being the only gene upregulated ≥ 2-fold in all four assayed conditions. Cell cycle upregulation is associated with the E2F/RB1 network; inactivation of RB1 due to mutation results in higher levels of specific FA genes. Importantly, our studies also indicate that *RB1*-mutated retinoblastoma are not characterized by a general upregulation of the canonical FA pathway, since the important core complex member *FANCF* turned out to be lower expressed associated with low DNA copy numbers; a finding with direct therapeutic implications. Surprisingly, two tools exploiting public data sets indicated coregulation of *FANCA* (acting in the upstream branch of the FA network) with *BRCA1* (acting downstream).

The encoded proteins of coregulated genes often participate in the same pathway. When we characterized the expression of genes of the FA/BRCA-pathway, we found evidence for highly divergent cell type and stimulus-dependent regulation of mRNA levels for the FA pathway, which only can become apparent by in parallel comparisons as done in this study. Especially, the genes encoding for proteins which build up the core complex show strikingly different levels of cell cycle regulation. When comparing the individual subcomplexes from the core complex, one member of a subcomplex turned out to be stronger cell cycle regulated than the other member(s), e.g. FANCA versus FANCG (Figure 1b). Interestingly, the more strongly cell cycle regulated gene of each subcomplex contains Nuclear Localization Signal (NLS)-encoding sequences, while the other partner(s) lacks a positive motif score (Haitjema et al. 2013). It could be hypothesized that the FA genes/proteins of the core complex bearing NLSs constitute the driving forces for nuclear complex assembly. Overall, in our assays, *FANCA* turned out to be most affected by proliferative stimuli. In line with this, *FANCA* induction had also been observed employing two other cell-cycle approaches (Whitfield et al. 2002; Hoskins et al. 2008). Together, this provides evidence for strong cell cycle regulation of especially *FANCA*.

As could be expected, there is an involvement of the RB1/E2F pathway in the regulation of FA genes, as had already been noted for selected FA genes (Tategu et al. 2007; Hoskins et al. 2008). Here, we focused on the FA/BRCA gene expression in cancers with disrupted RB1/E2F pathway, which further underscored the interplay of both pathways. Moreover, we showed that in *MYCN*-amplified retinoblastoma, with an intact RB1/E2F pathway, *FANCA* (and also *BRCA1*) are downregulated compared to *RB1*-mutated retinoblastoma. *FANCA* was also found to be upregulated in basal breast tumors compared to not-basal breast tumors.

We as well show that activating E2F genes are associated with upregulation of FA genes. Intriguingly, overexpression of *E2F3* strongly correlates with a high expression of *FANCA* and *BRCA1* in *RB1-*mutated retinoblastoma, while in *MYCN*-amplified retinoblastoma all three genes show relatively low expression. *FANCA* and *BRCA1* had already been denoted as E2F3-target genes in two other settings, while importantly no other FA/BRCA genes were identified (Polager et al. 2002; Bild et al. 2006). However, also other E2F genes likely contribute to the activation of FA genes (Tategu et al. 2007; Hoskins et al. 2008).

In all, no evidence was found for coordinate upregulation of the canonical FA pathway in response to proliferative stimuli. Due to low levels of the *FANCF* gene, the FA/BRCA-pathway may in fact be hypo-activated in full-blown retinoblastoma. Downregulation of *FANCF* mRNA levels in primary *RB1*-mutated retinoblastoma had been noted before (Ganguly and Shields 2010). Hyperactivation of the FA/BRCA-pathway has been associated with resistance against certain drugs including melphalan, while repression of the FA/BRCA-pathway has been linked with sensitivity. The combination of upregulation of specific FA genes with low *FANCF* expression and therefore likely low FANCF protein might explain the intermediate melphalan sensitivity observed in retinoblastoma cell lines [unpublished data], and why melphalan treatment can be successful in a subset of retinoblastoma patients (Venturi et al. 2013). Low levels of *FANCF* gene expression correlated with low levels of DNA was also found in basal breast cancer. Downregulation of *FANCF* due to hypermethylation of the *FANCF* promoter has also been reported in other tumors (Tischkowitz et al. 2003; Narayan et al. 2004; Wreesmann et al. 2007). The inactivation and/or low levels of FANCF which therefore disrupts the canonical FA pathway might also enhance alternative routes/functions of FA proteins, such as e.g. a FANCA-BRCA1 subcomplex (Folias et al. 2002).

**Figure 4:**
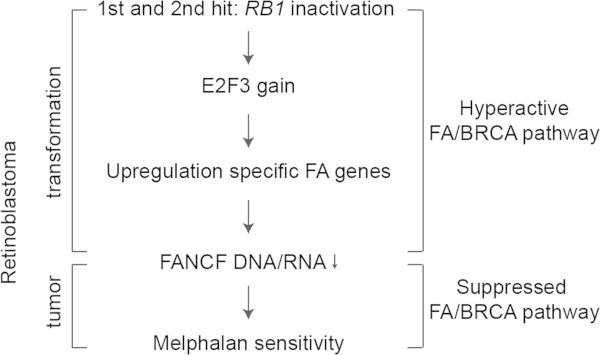
**Retinoblastoma and FA/BRCA pathway transformation model.** First and second hits in retinoblastoma are the inactivation of the RB1 alleles. Gain of E2F3 copy numbers aids to the upregulation of specific FA genes. In later stage inactivation of the FA/BRCA-pathway may be accomplished via low levels of the FANCF DNA/RNA, resulting in melphalan sensitivity.

In retinoblastoma upregulation of FA genes is an early event, since the first and second hit evolves mutations in the *RB1* gene. In addition, gain in copy numbers of E2F3 have already been found in retinoma, which is currently regarded as the precursor of retinoblastoma (Sampieri et al. 2008). It could be hypothesized, that in early stages FA pathway activation may aid tumorigenesis. The low FANCF levels observed in full-blown tumors, nevertheless, suggest that at later stages there may be a selection against hyperactivation of the FA pathway. This order is in accordance with a recent model described for the action of the transforming human papillomavirus (HPV) E7 protein. The FA pathway has been shown to be upregulated upon HPV infection (the transforming HPV protein E7 binds to RB1 releasing E2F to transactivate its targets), though the same activated FA pathway limits the accumulation of E7 - and thereby infection or transformation - via an unknown mechanism (Hoskins et al. 2012). Therefore, both in viral and non-viral *RB1*-linked cancers, an early general upregulation of the FA network may be followed by a dampening of the same network via other mechanisms (Figure 4). Upregulation of FA genes could be related to prevention of replication stress in which the FA/BRCA pathway plays a role (Schlacher et al. 2012). Reduction of replication stress at certain times of tumorigenesis might reduce DNA-damage induced apoptosis thereby facilitating tumorigenesis (Hills and Diffley 2014).

Our analysis suggests also the possible relevance of subnetworks of the FA pathway in pathology. Two tools exploiting public data sets indicated coregulation of *FANCA* - functioning in the upstream branch of the classic FA network - with *BRCA1* functioning in the downstream branch. In fact, coregulation of *FANCA* and *BRCA1* was also observed in studies on the transcription factor SMAD4 (Bornstein et al. 2009; Meier and Schindler 2011). In our previous published prioritization approach based on FA protein properties, BRCA1 and FANCA were number one and three, respectively (Haitjema et al. 2013); resembling also each other on multiple intrinsic protein properties.

Additional analysis of genes co-expressed with *FANCA* and *BRCA1*, showed enrichment for the cellular component centrosome. In line with this, FANCA and BRCA1, but also the inducing transcription factor E2F3, have all three been associated with centrosome function (Saavedra et al. 2003; Kais et al. 2012; Kim et al. 2013); loss gives rise to aberrant centrosomes. Importantly, in BRCA1 this function could be separated from its function in homologous recombination. In accordance with this, in uterine leiomyomas *FANCA* and *BRCA1* are both lowly expressed (Cirilo et al. 2013), and these tumors display centrosome dysfunction (Shan et al. 2012). Intriguingly, centrosome amplification causes microcephaly (Marthiens et al. 2013), a MeSH term we find enriched in the co-expressed genes.

BRCA1 has not been considered an FA gene, until recently, when a female patient diagnosed with ovarian cancer appeared to harbour biallelic mutations in *BRCA1* and should be regarded as a FA patient (Domchek et al. 2013). The affected individual presented with microcephaly and, as expected, was extremely sensitive to cross-linking drugs. Since BRCA1 and FANCA have been shown to interact (Folias et al. 2002), this warrants further study to explore how precisely FANCA and BRCA1 interact.

In summary, FA genes show a variable level of cell cycle regulation. Upregulation of a subset of genes of the FA network may be a common theme in *RB1*-mutated tumors. There may be a selection, however, against hyperactivation of the classic FA pathway in certain tumors. Furthermore, this study warrants further molecular studies of subcomplexes containing specific FA proteins, besides the canonical FA pathway, and their relevance for pathology, carcinogenesis and therapy response.

## 4 Methods

### 4.1 Cell and culture conditions

T98G glioblastoma cells (obtained from ATCC®) were cultured in Dulbecco’s modified Eagle medium (DMEM) containing 1 g/L D-Glucose supplemented with 10% foetal bovine serum (FBS). EVA-F primary diploid fibroblasts (obtained from a 23-year old female control; established and propagated at VUmc) were cultured in DMEM containing 4.5 g/L D-Glucose supplemented with 10% foetal bovine serum (FBS). To induce growth arrest, subconfluent cells were grown for 72 h in DMEM supplemented with 0.2% FBS. Cells were harvested at specific time points after restimulation with 10% FBS. Cell cycle analysis was performed as described previously (Stoepker et al. 2011). For EVA-F fibroblast, the karyotype was determined (EVA-F: 45.18 ± SEM 0.25 chromosomes, n = 40 metaphases).

### 4.2 RT-PCR and quantitative real-time PCR

Total RNA was extracted (High Pure Isolation Kit; Roche) and cDNA was prepared (iScriptcDNA Synthesis Kit; Biorad). The mRNA levels were quantified by real-time quantitative polymerase chain reactions (SYBR Green reaction kit; LightCycler 480, Roche). Relative gene expression, in terms of mean fold changes (MFC), was calculated via the 2^-ΔΔC^_T_ method (Livak and Schmittgen 2001). Primer design was optimized for quantitative mRNA profiling. The primers generated amplicons of ~100 to 250 bp with a minimum GC content of 40%; forward and reverse primers were on different exons to avoid genomic products (except for *FANCF* which has only on exon). As controls, primer sets for the established cell cycle marker gene *CCNE2* and for the housekeeping gene *TBP* were used. Specific primer pairs were designed using the Primer3 program (Primer3 Version 0.4.0; http://bioinfo.ut.ee/primer3-0.4.0/primer3/) for the genes as follows:

- *FANCA*: 5′-CACACGCTTGGCAGTGTAAT-3′ and 5′-CGCAAAGCTCCACTCTCTCT-3′;
- *FANCB*: 5′-CGCTGCGTTGAGTTTCATAA-′3 and 5′-TGGGACAATAGGCATCACAA-3′;
- *FANCC*: 5′-ATTCCGGGTTGTTGATGAGA-3′and 5′-TGCTTGCTTGCTTTCTCCAG-3′;
- *BRCA2/FANCD1*: 5′-ATGGCTCATACCCTCCAATG-3′ and 5′-TTCCATAGCTGCCAGTTTCC-3′;
- *FANCD2*: 5′-TCCGACTTGACCCAAACTTC-3′ and 5′-GTGATGGCAAAACACAATGC-3′;
- *FANCE*: 5′-TGATCTCAGCCTCAGCAATG-3′ and 5′-GGAGGTCAGGGCAGTTGTAA-3′;
- *FANCF*: 5′-CTAACTGCCCTGGAGACCTG-3′ and 5′-CGCTGAGACCCAAAACTTGT-3′;
- *FANCG*: 5′-CGCCCTAATTAGTCGTGGAC-3′ and 5′-TCCCTCCGATCTAGCCTCTT-3′;
- *FANCI*: 5′-AAGCGGGTAAAGCCAAAACT-3′ and 5′-CGCATAAACTCATTGCTGGA-3′;
- *BRIP1/FANCJ*: 5′-GCTCTCAGAAGTCGGTTTCC-3′ and 5′-AGCAAGCTGTGACGGGTAAG-3′;
- *FANCL*: 5′-GAAATTGATTTTCCAGCTCGTG-3′ and 5′-TGGTACCGTCAAGTTGATAAGC-3′;
- *FANCM*: 5′-CACGAAGGGTTTTACCCAGA-3′ and 5′-ACCTTCTTCACCCACACAGG-3′;
- *PALB2/FANCN*: 5′-CTTGGCCTGACAAAGAGGAG-3′ and 5′-AAGCAGAGCTTCTTGCATCC-3′;
- *BRCA1*: 5′-GAGTGAACCCGAAAATCCTTC-3′ and 5′-ACTGATTTCATCCCTGGTTCC-3′;
- *CCNE2*: 5′-ACTGACTGCTGCTGCCTTGTGC-3′ and 5′-TCGGTGGTGTCATAATGCCTCC-3′;
- *TBP*: 5′-TGCACAGGAGCCAAGAGTGAA-3′and 5′-CACATCACAGCTCCCCACCA-3′.

### 4.3 Gene expression profiling

#### 4.3.1 Primary retinoblastoma tumors

Total RNA was isolated from 77 primary retinoblastoma tumors and 3 healthy fetal retina tissues (including 2 biological duplicates adding up to 5 control RNA-extracts). Biotinylated targets were prepared by published methods (Lipshutz et al. 1999) and hybridized to Affymetrix HT HG-U133 Plus PM arrays. Resulting raw CEL-files were normalized by robust multi-array average implementated by Bioconductor package affy (Gautier et al. 2004). To identify genes differentially expressed between the retinoblastoma tumors and healthy fetal retina, empirical Bayes moderated t-statistics were calculated, implemented by the limma package (Smyth 2005) and p-values were adjusted by Benjamini and Hochberg multiple testing correction (Benjamini and Hochberg 1995).

#### 4.3.2 Basal and not-basal breast tumors

Total RNA was isolated from 41 basal breast tumors (27 *BRCA1* mutated) and 79 not-basal breast tumors (8 *BRCA1* mutated) and cryostat sections using RNAzol B (Campro Scientific, Veenendaal, The Netherlands) and RNA quality and quantity was evaluated on a Agilent Bioanalyzer. Antisense biotinylated RNA was prepared and hybridized to Affymetrix HGU133_Plus_2.0 GeneChips, according to the manufacturer’s guidelines (Affymetrix, Santa Clara, CA, USA). Gene expression signals were calculated using AffymetrixGeneChip analysis software MAS 5.0. Global scaling normalization was performed to bring the average signal intensity of the chips to a target of 100 before data analysis. The data was imported in Partek Genomics Suite 6.5 and Log2 transformed before analysis. ANOVA was then used to determine differentially expressed probe-sets.

### 4.4 Analysis of public data sets and gene set enrichment analysis

Two online annotation tools were used for in silico coregulation analysis: BioGPS (Wu et al. 2009) and GeneFriends (http://genefriends.org/microArray/) (van Dam et al. 2012). With BioGPS the data set GeneAtlas U133A, gcrma was analyzed, with the cut-off: >0.7 (Su et al. 2004). The human co-expression network was screened in GeneFriends. Enrichment analysis was performed with Genomatix GeneRanker (Genomatix Software GmbH, version 2013).

### 4.5 Ethical standards

Retinoblastoma and breast cancer specimens were processed and analyzed in accordance with local ethics (VUmc).

## Electronic supplementary material

Additional file 1: Figure S1: Differential cell cycle regulation of FA genes in human EVA-F cells. (a - left panel) Cells were placed on medium with low serum (0.2% FBS) for 3 days resulting in cell cycle arrest. The addition of high serum medium (10% FBS) released cells resulting in synchronous progression through the cell cycle. Sampled cells at different time points were divided for Fluorescent Activated Cell Sorting (FACS) analysis. Data represents one representative synchronization experiment. Quantitative RT-PCR was performed on RNA samples from different time points and mean fold changes (MFC) were calculated relative to time point zero. Data represents duplo qPCR measurements of one representative synchronization experiment, SEM is indicated. (a - right panel) Cell cycle control *CCNE2* (b) *FANCA* and *FANCG* (c) *FANCB* and *FANCL* (d) *FANCE*, *FANCC*, and *FANCF* (e) *FANCM* (f) *FANCD2* and *FANCI* (g) *BRCA2, PALB2, BRCA1,* and *BRIP1*. (TIFF 612 KB)

Additional file 2: Table S1: Expression in retinoblastoma tumors with *MYCN* amplification versus retinoblastoma tumors with *RB1* mutations. **Table S2** Summary regulation FA/BRCA-pathway genes cell model. **Table S3** Summary results FA/BRCA-pathway genes “RB1/E2F disturbed cells”. **Table S4** Co-expressed genes with input gene *FANCA*. **Table S5** Co-expressed genes with input gene *FANCA* and *BRCA1*. **Table S6** Medical Subject Headings (MeSH) enrichment of 50 genes co-expressed with *FANCA* and *BRCA1*. **Table S7** Cellular Components (GO) enrichment of 50 genes co-expressed with *FANCA* and *BRCA1*. (DOC 210 KB)

## Abbreviations

CNA: Copy Number Alteration
DMEM: Dulbecco’s modified Eagle medium
DSB: Double strand break
FA: Fanconi anemia
FACS: Fluorescent Activated Cell Sorting
FBS: Foetal bovine serum
FC: Fold Change
FDR: False Discovery Rate
MeSH: Medical Subject Heading
MFC: Mean Fold Change
RB: Retinoblastoma
SEM: Standard Error of the Mean.

## References

[CR1] Alter BP (2003). Cancer in Fanconi anemia, 1927-2001. Cancer.

[CR2] Apostolou S, Whitmore SA, Crawford J, Lennon G, Sutherland GR, Callen DF, Lanzano L, Savino M, D’Apolito M, Notarangeio A, Memeo E, Piemontese MR, Zelante L, Savoia A, Gibson RA, Tipping AJ, Morgan NV, Hassock S, Jansen S, de Ravel TJ, Van Berkell C, Pronk JC, Easton DF, Mathew CG, Levran O, Verlander PC, Batish SD, Erlich T, Auerbach AD, Cleton-Jansen A-M, Moerland EW, Cornelisse CJ, Doggett NA, Deaven LL, Moyzis RK (1996). Positional cloning of the Fanconi anaemia group A gene. Nat Genet.

[CR3] Auerbach A, Buchwald M, Joenje H (2001). Fanconi anemia. The metabolic and molecular bases of inherited disease.

[CR4] Benjamini Y, Hochberg Y (1995). Controlling the False Discovery Rate: A Practical and Powerful Approach to Multiple Testing. Journal of the Royal Statistical Society Series B (Methodological).

[CR5] Bild AH, Yao G, Chang JT, Wang Q, Potti A, Chasse D, Joshi M-B, Harpole D, Lancaster JM, Berchuck A, Olson JA, Marks JR, Dressman HK, West M, Nevins JR (2006). Oncogenic pathway signatures in human cancers as a guide to targeted therapies. Nature.

[CR6] Bogliolo M, Schuster B, Stoepker C, Derkunt B, Su Y, Raams A, Trujillo JP, Minguillón J, Ramírez MJ, Pujol R, Casado JA, Baños R, Rio P, Knies K, Zúñiga S, Benítez J, Bueren JA, Jaspers NGJ, Schärer OD, de Winter JP, Schindler D, Surrallés J (2013). Mutations in ERCC4, Encoding the DNA-Repair Endonuclease XPF, Cause Fanconi Anemia. Am J Hum Genet.

[CR7] Bornstein S, White R, Malkoski S, Oka M, Han G, Cleaver T, Reh D, Andersen P, Gross N, Olson S, Deng C, Lu S-L, Wang X-J (2009). Smad4 loss in mice causes spontaneous head and neck cancer with increased genomic instability and inflammation. J Clin Invest.

[CR8] Chen Q, der Sluis PCV, Boulware D, Hazlehurst LA, Dalton WS (2005). The FA/BRCA pathway is involved in melphalan-induced DNA interstrand cross-link repair and accounts for melphalan resistance in multiple myeloma cells. Blood.

[CR9] Chen H-Z, Tsai S-Y, Leone G (2009). Emerging roles of E2Fs in cancer: an exit from cell cycle control. Nat Rev Cancer.

[CR10] Cirilo PDR, Marchi FA, de Camargo Barros Filho M, Rocha RM, Domingues MAC, Jurisica I, Pontes A, Rogatto SR (2013). An integrative genomic and transcriptomic analysis reveals potential targets associated with cell proliferation in uterine leiomyomas. PLoS ONE.

[CR11] D’Andrea AD (2013). BRCA1: A Missing Link in the Fanconi Anemia/BRCA Pathway. Cancer Discovery.

[CR12] De Winter JP, Joenje H (2009). The genetic and molecular basis of Fanconi anemia. Mutat Res.

[CR13] De Winter JP, Waisfisz Q, Rooimans MA, van Berkel CG, Bosnoyan-Collins L, Alon N, Carreau M, Bender O, Demuth I, Schindler D, Pronk JC, Arwert F, Hoehn H, Digweed M, Buchwald M, Joenje H (1998). The Fanconi anaemia group G gene FANCG is identical with XRCC9. Nat Genet.

[CR14] De Winter JP, Léveillé F, van Berkel CG, Rooimans MA, van Der Weel L, Steltenpool J, Demuth I, Morgan NV, Alon N, Bosnoyan-Collins L, Lightfoot J, Leegwater PA, Waisfisz Q, Komatsu K, Arwert F, Pronk JC, Mathew CG, Digweed M, Buchwald M, Joenje H (2000). Isolation of a cDNA representing the Fanconi anemia complementation group E gene. Am J Hum Genet.

[CR15] De Winter JP, Rooimans MA, van Der Weel L, van Berkel CG, Alon N, Bosnoyan-Collins L, de Groot J, Zhi Y, Waisfisz Q, Pronk JC, Arwert F, Mathew CG, Scheper RJ, Hoatlin ME, Buchwald M, Joenje H (2000). The Fanconi anaemia gene FANCF encodes a novel protein with homology to ROM. Nat Genet.

[CR16] Dear PH (2009). Copy-number variation: the end of the human genome?. Trends in Biotechnology.

[CR17] Di Fiore R, D’Anneo A, Tesoriere G, Vento R (2013). RB1 in cancer: different mechanisms of RB1 inactivation and alterations of pRb pathway in tumorigenesis. J Cell Physiol.

[CR18] Domchek SM, Tang J, Stopfer J, Lilli DR, Hamel N, Tischkowitz M, Monteiro AN, Messick TE, Powers J, Yonker A, Couch FJ, Goldgar DE, Davidson HR, Nathanson KL, Foulkes WD, Greenberg RA (2013). Biallelic deleterious BRCA1 mutations in a woman with early-onset ovarian cancer. Cancer Discov.

[CR19] Dorsman JC, Levitus M, Rockx D, Rooimans MA, Oostra AB, Haitjema A, Bakker ST, Steltenpool J, Schuler D, Mohan S, Schindler D, Arwert F, Pals G, Mathew CG, Waisfisz Q, de Winter JP, Joenje H (2007). Identification of the Fanconi anemia complementation group I gene, FANCI. Cell Oncol.

[CR20] Dunn JM, Phillips RA, Becker AJ, Gallie BL (1988). Identification of germline and somatic mutations affecting the retinoblastoma gene. Science.

[CR21] Folias A, Matkovic M, Bruun D, Reid S, Hejna J, Grompe M, D’Andrea A, Moses R (2002). BRCA1 interacts directly with the Fanconi anemia protein FANCA. Hum Mol Genet.

[CR22] Ganguly A, Shields CL (2010). Differential gene expression profile of retinoblastoma compared to normal retina. Mol Vis.

[CR23] Gautier L, Cope L, Bolstad BM, Irizarry RA (2004). affy–analysis of Affymetrix GeneChip data at the probe level. Bioinformatics.

[CR24] Haitjema A, Brandt BW, Ameziane N, May P, Heringa J, de Winter JP, Joenje H, Dorsman JC (2013). A Protein Prioritization Approach Tailored for the FA/BRCA Pathway. PLoS ONE.

[CR25] Henley SA, Dick FA (2012). The retinoblastoma family of proteins and their regulatory functions in the mammalian cell division cycle. Cell Div.

[CR26] Henrichsen CN, Chaignat E, Reymond A (2009). Copy number variants, diseases and gene expression. Hum Mol Genet.

[CR27] Herschkowitz JI, He X, Fan C, Perou CM (2008). The functional loss of the retinoblastoma tumour suppressor is a common event in basal-like and luminal B breast carcinomas. Breast Cancer Res.

[CR28] Hills SA, Diffley JFX (2014). DNA Replication and Oncogene-Induced Replicative Stress. Curr Biol.

[CR29] Hoskins EE, Gunawardena RW, Habash KB, Wise-Draper TM, Jansen M, Knudsen ES, Wells SI (2008). Coordinate regulation of Fanconi anemia gene expression occurs through the Rb/E2F pathway. Oncogene.

[CR30] Hoskins EE, Morreale RJ, Werner SP, Higginbotham JM, Laimins LA, Lambert PF, Brown DR, Gillison ML, Nuovo GJ, Witte DP, Kim M-O, Davies SM, Mehta PA, Butsch Kovacic M, Wikenheiser-Brokamp KA, Wells SI (2012). The fanconi anemia pathway limits human papillomavirus replication. J Virol.

[CR31] Howlett NG, Taniguchi T, Olson S, Cox B, Waisfisz Q, De Die-Smulders C, Persky N, Grompe M, Joenje H, Pals G, Ikeda H, Fox EA, D’Andrea AD (2002). Biallelic inactivation of BRCA2 in Fanconi anemia. Science.

[CR32] Ishida R, Buchwald M (1982). Susceptibility of Fanconi’s anemia lymphoblasts to DNA-cross-linking and alkylating agents. Cancer Res.

[CR33] Kaddar T, Carreau M (2012). Fanconi anemia proteins and their interacting partners: a molecular puzzle. Anemia.

[CR34] Kais Z, Chiba N, Ishioka C, Parvin JD (2012). Functional differences among BRCA1 missense mutations in the control of centrosome duplication. Oncogene.

[CR35] Kim H, D’Andrea AD (2012). Regulation of DNA cross-link repair by the Fanconi anemia/BRCA pathway. Genes Dev.

[CR36] Kim Y, Lach FP, Desetty R, Hanenberg H, Auerbach AD, Smogorzewska A (2011). Mutations of the SLX4 gene in Fanconi anemia. Nat Genet.

[CR37] Kim S, Hwang SK, Lee M, Kwak H, Son K, Yang J, Kim SH, Lee C-H (2013). Fanconi anemia complementation group A (FANCA) localizes to centrosomes and functions in the maintenance of centrosome integrity. Int J Biochem Cell Biol.

[CR38] Knudsen ES, Wang JYJ (2010). Targeting the RB-pathway in Cancer Therapy. Clin Cancer Res.

[CR39] Kuiper RP, Ligtenberg MJL, Hoogerbrugge N, Geurts van Kessel A (2010). Germline copy number variation and cancer risk. Curr Opin Genet Dev.

[CR40] Kutler DI, Singh B, Satagopan J, Batish SD, Berwick M, Giampietro PF, Hanenberg H, Auerbach AD (2003). A 20-year perspective on the International Fanconi Anemia Registry (IFAR). Blood.

[CR41] Levitus M, Waisfisz Q, Godthelp BC, de Vries Y, Hussain S, Wiegant WW, Elghalbzouri-Maghrani E, Steltenpool J, Rooimans MA, Pals G, Arwert F, Mathew CG, Zdzienicka MZ, Hiom K, De Winter JP, Joenje H (2005). The DNA helicase BRIP1 is defective in Fanconi anemia complementation group J. Nat Genet.

[CR42] Levran O, Attwooll C, Henry RT, Milton KL, Neveling K, Rio P, Batish SD, Kalb R, Velleuer E, Barral S, Ott J, Petrini J, Schindler D, Hanenberg H, Auerbach AD (2005). The BRCA1-interacting helicase BRIP1 is deficient in Fanconi anemia. Nat Genet.

[CR43] Levran O, Diotti R, Pujara K, Batish SD, Hanenberg H, Auerbach AD (2005). Spectrum of sequence variations in the FANCA gene: An International Fanconi Anemia Registry (IFAR) study. Human Mutation.

[CR44] Lipshutz RJ, Fodor SP, Gingeras TR, Lockhart DJ (1999). High density synthetic oligonucleotide arrays. Nat Genet.

[CR45] Livak KJ, Schmittgen TD (2001). Analysis of relative gene expression data using real-time quantitative PCR and the 2(-Delta Delta C(T)) Method. Methods.

[CR46] Lo Ten Foe JR, Rooimans MA, Bosnoyan-Collins L, Alon N, Wijker M, Parker L, Lightfoot J, Carreau M, Callen DF, Savoia A, Cheng NC, van Berkel CG, Strunk MH, Gille JJ, Pals G, Kruyt FA, Pronk JC, Arwert F, Buchwald M, Joenje H (1996). Expression cloning of a cDNA for the major Fanconi anaemia gene, FAA. Nat Genet.

[CR47] Marthiens V, Rujano MA, Pennetier C, Tessier S, Paul-Gilloteaux P, Basto R (2013). Centrosome amplification causes microcephaly. Nat Cell Biol.

[CR48] Medhurst AL, Laghmani EH, Steltenpool J, Ferrer M, Fontaine C, de Groot J, Rooimans MA, Scheper RJ, Meetei AR, Wang W, Joenje H, de Winter JP (2006). Evidence for subcomplexes in the Fanconi anemia pathway. Blood.

[CR49] Meetei AR, de Winter JP, Medhurst AL, Wallisch M, Waisfisz Q, van de Vrugt HJ, Oostra AB, Yan Z, Ling C, Bishop CE, Hoatlin ME, Joenje H, Wang W (2003). A novel ubiquitin ligase is deficient in Fanconi anemia. Nat Genet.

[CR50] Meetei AR, Levitus M, Xue Y, Medhurst AL, Zwaan M, Ling C, Rooimans MA, Bier P, Hoatlin M, Pals G, de Winter JP, Wang W, Joenje H (2004). X-linked inheritance of Fanconi anemia complementation group B. Nat Genet.

[CR51] Meetei AR, Medhurst AL, Ling C, Xue Y, Singh TR, Bier P, Steltenpool J, Stone S, Dokal I, Mathew CG, Hoatlin M, Joenje H, de Winter JP, Wang W (2005). A human ortholog of archaeal DNA repair protein Hef is defective in Fanconi anemia complementation group M. Nat Genet.

[CR52] Meier D, Schindler D (2011). Fanconi anemia core complex gene promoters harbor conserved transcription regulatory elements. PLoS ONE.

[CR53] Narayan G, Arias-Pulido H, Nandula SV, Basso K, Sugirtharaj DD, Vargas H, Mansukhani M, Villella J, Meyer L, Schneider A, Gissmann L, Dürst M, Pothuri B, Murty VVVS (2004). Promoter hypermethylation of FANCF: disruption of Fanconi Anemia-BRCA pathway in cervical cancer. Cancer Res.

[CR54] Nevins JR (2001). The Rb/E2F pathway and cancer. Hum Mol Genet.

[CR55] Polager S, Kalma Y, Berkovich E, Ginsberg D (2002). E2Fs up-regulate expression of genes involved in DNA replication, DNA repair and mitosis. Oncogene.

[CR56] Pronk JC, Gibson RA, Savoia A, Wijker M, Morgan NV, Melchionda S, Ford D, Temtamy S, Ortega JJ, Jansen S (1995). Localisation of the Fanconi anaemia complementation group A gene to chromosome 16q24.3. Nat Genet.

[CR57] Rosenberg PS, Greene MH, Alter BP (2003). Cancer incidence in persons with Fanconi anemia. Blood.

[CR58] Rushlow DE, Mol BM, Kennett JY, Yee S, Pajovic S, Thériault BL, Prigoda-Lee NL, Spencer C, Dimaras H, Corson TW, Pang R, Massey C, Godbout R, Jiang Z, Zacksenhaus E, Paton K, Moll AC, Houdayer C, Raizis A, Halliday W, Lam WL, Boutros PC, Lohmann D, Dorsman JC, Gallie BL (2013). Characterisation of retinoblastomas without RB1 mutations: genomic, gene expression, and clinical studies. Lancet Oncol.

[CR59] Saavedra HI, Maiti B, Timmers C, Altura R, Tokuyama Y, Fukasawa K, Leone G (2003). Inactivation of E2F3 results in centrosome amplification. Cancer Cell.

[CR60] Sampieri K, Mencarelli MA, Epistolato MC, Toti P, Lazzi S, Bruttini M, De Francesco S, Longo I, Meloni I, Mari F, Acquaviva A, Hadjistilianou T, Renieri A, Ariani F (2008). Genomic differences between retinoma and retinoblastoma. Acta Oncol.

[CR61] Schlacher K, Wu H, Jasin M (2012). A Distinct Replication Fork Protection Pathway Connects Fanconi Anemia Tumor Suppressors to RAD51-BRCA1/2. Cancer Cell.

[CR62] Shan W, Akinfenwa PY, Savannah KB, Kolomeyevskaya N, Laucirica R, Thomas DG, Odunsi K, Creighton CJ, Lev DC, Anderson ML (2012). A small-molecule inhibitor targeting the mitotic spindle checkpoint impairs the growth of uterine leiomyosarcoma. Clin Cancer Res.

[CR63] Sims AE, Spiteri E, Sims RJ, Arita AG, Lach FP, Landers T, Wurm M, Freund M, Neveling K, Hanenberg H, Auerbach AD, Huang TT (2007). FANCI is a second monoubiquitinated member of the Fanconi anemia pathway. Nat Struct Mol Biol.

[CR64] Smogorzewska A, Matsuoka S, Vinciguerra P, McDonald ER, Hurov KE, Luo J, Ballif BA, Gygi SP, Hofmann K, D’Andrea AD, Elledge SJ (2007). Identification of the FANCI protein, a monoubiquitinated FANCD2 paralog required for DNA repair. Cell.

[CR65] Smyth GK, Gentleman R, Carey VJ, Huber W, Irizarry RA, Dudoit S (2005). limma: Linear Models for Microarray Data. Bioinformatics and Computational Biology Solutions Using R and Bioconductor.

[CR66] Stecklein SR, Jensen RA (2012). Identifying and exploiting defects in the Fanconi anemia/BRCA pathway in oncology. Transl Res.

[CR67] Stein GH (1979). T98G: an anchorage-independent human tumor cell line that exhibits stationary phase G1 arrest in vitro. J Cell Physiol.

[CR68] Stoepker C, Hain K, Schuster B, Hilhorst-Hofstee Y, Rooimans MA, Steltenpool J, Oostra AB, Eirich K, Korthof ET, Nieuwint AWM, Jaspers NGJ, Bettecken T, Joenje H, Schindler D, Rouse J, de Winter JP (2011). SLX4, a coordinator of structure-specific endonucleases, is mutated in a new Fanconi anemia subtype. Nat Genet.

[CR69] Strathdee CA, Duncan AM, Buchwald M (1992). Evidence for at least four Fanconi anaemia genes including FACC on chromosome 9. Nat Genet.

[CR70] Su AI, Wiltshire T, Batalov S, Lapp H, Ching KA, Block D, Zhang J, Soden R, Hayakawa M, Kreiman G, Cooke MP, Walker JR, Hogenesch JB (2004). A gene atlas of the mouse and human protein-encoding transcriptomes. Proc Natl Acad Sci USA.

[CR71] Takahashi Y, Rayman JB, Dynlacht BD (2000). Analysis of promoter binding by the E2F and pRB families in vivo: distinct E2F proteins mediate activation and repression. Genes Dev.

[CR72] Tategu M, Arauchi T, Tanaka R, Nakagawa H, Yoshida K (2007). Systems biology-based identification of crosstalk between E2F transcription factors and the Fanconi anemia pathway. Gene Regul Syst Bio.

[CR73] Timmers C, Taniguchi T, Hejna J, Reifsteck C, Lucas L, Bruun D, Thayer M, Cox B, Olson S, D’Andrea AD, Moses R, Grompe M (2001). Positional cloning of a novel Fanconi anemia gene, FANCD2. Mol Cell.

[CR74] Tischkowitz M, Ameziane N, Waisfisz Q, De Winter JP, Harris R, Taniguchi T, D’Andrea A, Hodgson SV, Mathew CG, Joenje H (2003). Bi-allelic silencing of the Fanconi anaemia gene FANCF in acute myeloid leukaemia. Br J Haematol.

[CR75] van Dam S, Cordeiro R, Craig T, van Dam J, Wood SH, de Magalhães JP (2012). GeneFriends: An online co-expression analysis tool to identify novel gene targets for aging and complex diseases. BMC Genomics.

[CR76] Vaz F, Hanenberg H, Schuster B, Barker K, Wiek C, Erven V, Neveling K, Endt D, Kesterton I, Autore F, Fraternali F, Freund M, Hartmann L, Grimwade D, Roberts RG, Schaal H, Mohammed S, Rahman N, Schindler D, Mathew CG (2010). Mutation of the RAD51C gene in a Fanconi anemia-like disorder. Nat Genet.

[CR77] Venturi C, Bracco S, Cerase A, Cioni S, Galluzzi P, Gennari P, Vallone IM, Tinturini R, Vittori C, De Francesco S, Caini M, D’Ambrosio A, Toti P, Renieri A, Hadjistilianou T (2013). Superselective ophthalmic artery infusion of melphalan for intraocular retinoblastoma: preliminary results from 140 treatments. Acta Ophthalmol.

[CR78] Waisfisz Q, Saar K, Morgan NV, Altay C, Leegwater PA, de Winter JP, Komatsu K, Evans GR, Wegner RD, Reis A, Joenje H, Arwert F, Mathew CG, Pronk JC, Digweed M (1999). The Fanconi anemia group E gene, FANCE, maps to chromosome 6p. Am J Hum Genet.

[CR79] Wang W (2007). Emergence of a DNA-damage response network consisting of Fanconi anaemia and BRCA proteins. Nat Rev Genet.

[CR80] Whitfield ML, Sherlock G, Saldanha AJ, Murray JI, Ball CA, Alexander KE, Matese JC, Perou CM, Hurt MM, Brown PO, Botstein D (2002). Identification of genes periodically expressed in the human cell cycle and their expression in tumors. Mol Biol Cell.

[CR81] Wilson JB, Yamamoto K, Marriott AS, Hussain S, Sung P, Hoatlin ME, Mathew CG, Takata M, Thompson LH, Kupfer GM, Jones NJ (2008). FANCG promotes formation of a newly identified protein complex containing BRCA2, FANCD2 and XRCC3. Oncogene.

[CR82] Wreesmann VB, Estilo C, Eisele DW, Singh B, Wang SJ (2007). Downregulation of Fanconi anemia genes in sporadic head and neck squamous cell carcinoma. ORL J Otorhinolaryngol Relat Spec.

[CR83] Wu C, Orozco C, Boyer J, Leglise M, Goodale J, Batalov S, Hodge CL, Haase J, Janes J, Huss JW, Su AI (2009). BioGPS: an extensible and customizable portal for querying and organizing gene annotation resources. Genome Biology.

[CR84] Xia B, Dorsman JC, Ameziane N, de Vries Y, Rooimans MA, Sheng Q, Pals G, Errami A, Gluckman E, Llera J, Wang W, Livingston DM, Joenje H, de Winter JP (2007). Fanconi anemia is associated with a defect in the BRCA2 partner PALB2. Nat Genet.

