# Supplementary figures and images for: Coregulation of FANCA and BRCA1 in human cells
Source: Springerplus. 2014 Jul 28;3:381. doi: 10.1186/2193-1801-3-381 (PMC4143540; doi:10.1186/2193-1801-3-381)

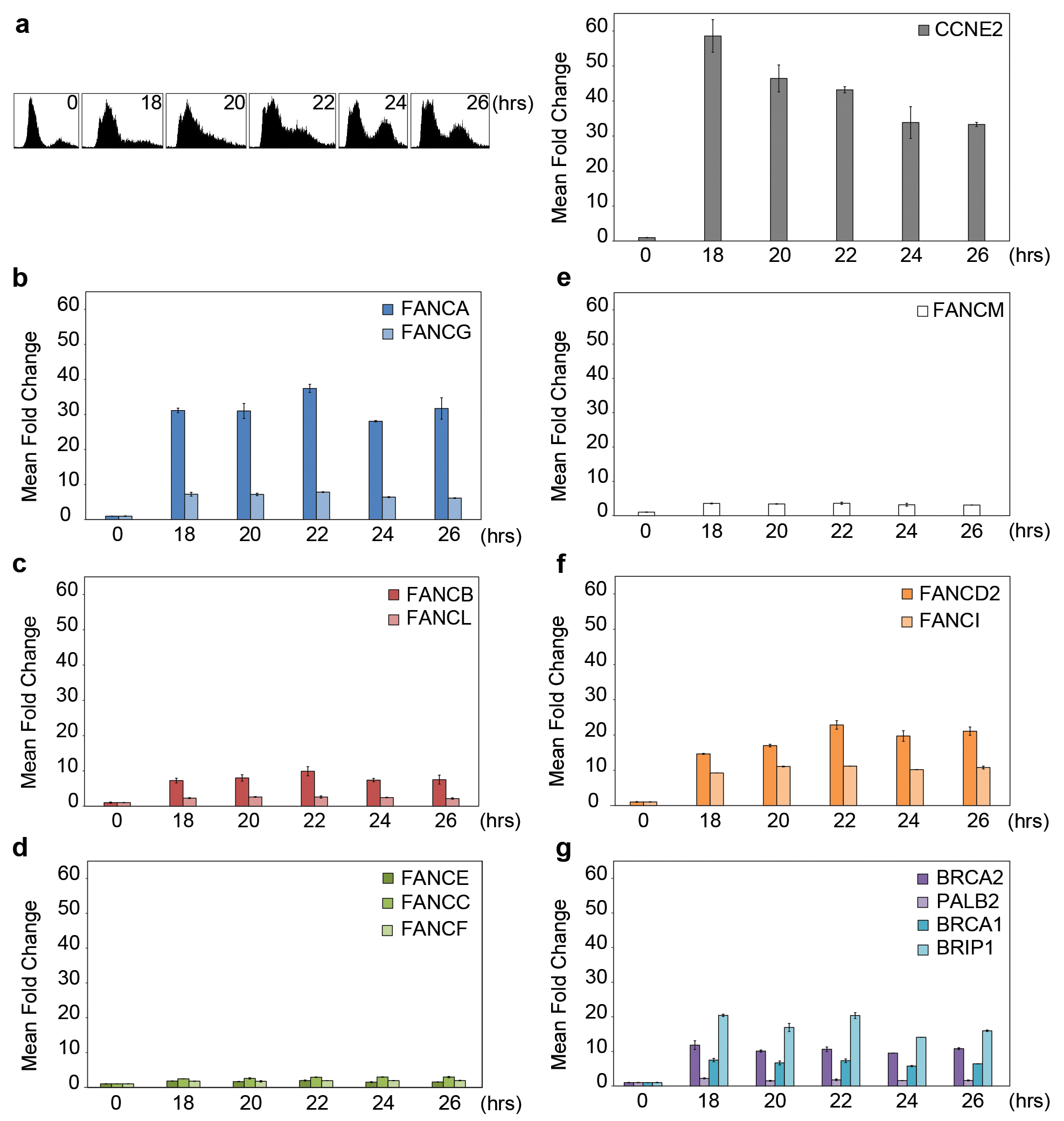

Supplement: Supplementary file 1 — Additional file 1: Figure S1: Differential cell cycle regulation of FA genes in human EVA-F cells. (a - left panel) Cells were placed on medium with low serum (0.2% FBS) for 3 days resulting in cell cycle arrest. The addition of high serum medium (10% FBS) released cells resulting in synchronous progression through the cell cycle. Sampled cells at different time points were divided for Fluorescent Activated Cell Sorting (FACS) analysis. Data represents one representative synchronization experiment. Quantitative RT-PCR was performed on RNA samples from different time points and mean fold changes (MFC) were calculated relative to time point zero. Data represents duplo qPCR measurements of one representative synchronization experiment, SEM is indicated. (a - right panel) Cell cycle control CCNE2 (b) FANCA and FANCG (c) FANCB and FANCL (d) FANCE, FANCC, and FANCF (e) FANCM (f) FANCD2 and FANCI (g) BRCA2, PALB2, BRCA1, and BRIP1. (TIFF 612 KB) [file 40064_2014_1142_MOESM1_ESM.tiff]
