# Supplementary material for: Coregulation of FANCA and BRCA1 in human cells
Source: Springerplus. 2014 Jul 28;3:381. doi: 10.1186/2193-1801-3-381 (PMC4143540; doi:10.1186/2193-1801-3-381)
Supplement: Supplementary file 2 — Additional file 2: Table S1: Expression in retinoblastoma tumors with MYCN amplification versus retinoblastoma tumors with RB1 mutations. Table S2 Summary regulation FA/BRCA-pathway genes cell model. Table S3 Summary results FA/BRCA-pathway genes “RB1/E2F disturbed cells”. Table S4 Co-expressed genes with input gene FANCA. Table S5 Co-expressed genes with input gene FANCA and BRCA1. Table S6 Medical Subject Headings (MeSH) enrichment of 50 genes co-expressed with FANCA and BRCA1. Table S7 Cellular Components (GO) enrichment of 50 genes co-expressed with FANCA and BRCA1. (DOC 210 KB) [file 40064_2014_1142_MOESM2_ESM.doc]

**Additional file 1 consisting of:**

**Additional Tables and Legend to Additional Figure S1**

**Coregulation of *FANCA* and *BRCA1* in human cells**

**SpringerPlus**

Anneke Haitjema, Berber M. Mol*, Irsan E. Kooi*, Maarten P. G. Massink*, Jens A.L. Jørgensen, Davy A.P. Rockx, Martin A. Rooimans, Johan P. de Winter †, Hanne Meijers-Heijboer, Hans Joenje, Josephine C. Dorsman^§^

Department of Clinical Genetics, VU University Medical Center, Amsterdam, The Netherlands

*Contributed equally

**^§^**To whom correspondence should be addressed: Dr. Josephine C. Dorsman, E-mail: jc.dorsman@vumc.nl

**Additional Tables**

**Table S1**

Expression in retinoblastoma tumors with *MYCN* amplification versus retinoblastoma tumors with *RB1* mutations

| **Gene Symbol** | **Probe ID** | **Fold Change** | **P-value^*^** |
| --- | --- | --- | --- |
| FANCA | 236976_PM_at | -3.99 | 3.69E-03 |
| FANCC | 205189_PM_s_at | -1.91 | 1.46E-02 |
| FANCI | 213007_PM_at | -3.37 | 3.55E-02 |
| FANCI | 213008_PM_at | -3.74 | 1.56E-02 |
| FANCL | 218397_PM_at | -2.56 | 2.72E-06 |
| FANCM | 1554277_PM_s_at | -2.07 | 2.16E-02 |
| BRCA1 | 204531_PM_s_at | -2.36 | 8.85E-03 |
| BRCA1 | 211851_PM_x_at | -2.06 | 4.61E-02 |
| E2F1 | 2028_PM_s_at | -2.28 | 3.65E-02 |
| E2F2 | 228361_PM_at | -3.39 | 7.31E-03 |

^*^Cutoff P-value < 0.05

**Table S2**

Summary regulation FA/BRCA-pathway genes cell model

|  |  | Regulated | |
| --- | --- | --- | --- |
|  |  | MFC > 2.0^*^ | |
| Part | Gene | T98G | EVA-F |
| Core complex | FANCA | x | x |
|  | FANCB | x | x |
|  | FANCC |  | x |
|  | FANCE | x |  |
|  | FANCF |  |  |
|  | FANCG | x | x |
|  | FANCL |  | x |
|  | FANCM | x | x |
| Central players | FANCD2 | x | x |
|  | FANCI | x | x |
| Downstrem branch | FANCD1/BRCA2 | x | x |
|  | FANCN/PALB2 | x | x |
|  | BRCA1 | x | x |
|  | FANCJ/BRIP1 | x | x |
| Control | CCNE2 | x | x |

**^*^**See also Figure 2a

**Table S3**

Summary results FA/BRCA-pathway genes “RB1/E2F disturbed cells”^*^

|  |  | **Retinoblastoma** | | | **Breast** | | |
| --- | --- | --- | --- | --- | --- | --- | --- |
| **Part** | **Gene Symbol** | **FC > 2** | **FC < 2** | **No Info** | **FC > 2** | **FC < 2** | **No Info** |
| Core complex | FANCA | x |  |  | x |  |  |
|  | FANCB |  |  | P > 0.05 | x |  |  |
|  | FANCC |  | x |  |  | x |  |
|  | FANCE |  | x^CNA^ |  |  | x |  |
|  | FANCF |  | x |  |  | x^CNA^ |  |
|  | FANCG | x |  |  |  | x |  |
|  | FANCL | x |  |  |  | x |  |
|  | FANCM |  | x |  |  | x |  |
| Central players | FANCD2 |  | x |  |  | x |  |
|  | FANCI | x |  |  |  | x^CNA^ |  |
| Downstrem branch | FANCD1/BRCA2 | x |  |  |  | x |  |
|  | FANCN/PALB2 | x |  |  |  |  | P > 0.05 |
|  | BRCA1 | x |  |  |  |  | # |
|  | FANCJ/BRIP1 |  |  | P > 0.05 | x |  |  |
| Acitvating E2Fs | E2F1 |  | x |  |  | x |  |
|  | E2F2 | x |  |  |  | x |  |
|  | E2F3 | x^CNA^ |  |  | x^CNA^ |  |  |
| Control | CCNE2 | x |  |  |  |  | P > 0.05 |

^*^See also Figure 2b

# Exception since both groups (basal and not-basal) contain *BRCA1* mutations.

**Table S4**

Co-expressed genes with input gene *FANCA*

| **Gene Symbol** | **Co-expression value** | **Total friends*** |
| --- | --- | --- |
| **FANCA** | **1** | **395** |
| ESPL1 | 0.75 | 2239 |
| POLQ | 0.746 | 1977 |
| MKI67 | 0.737 | 2338 |
| EZH2 | 0.735 | 3200 |
| GINS1 | 0.734 | 3494 |
| RAD54L | 0.733 | 1339 |
| OIP5 | 0.731 | 4483 |
| ASF1B | 0.73 | 2335 |
| MCM4 | 0.729 | 2645 |
| SPAG5 | 0.729 | 2491 |
| TPX2 | 0.729 | 2660 |
| BUB1B | 0.729 | 3501 |
| MYBL2 | 0.728 | 2597 |
| MCM2 | 0.727 | 2989 |
| RRM2 | 0.727 | 3029 |
| BIRC5 | 0.727 | 2442 |
| KIF15 | 0.727 | 2626 |
| KIFC1 | 0.726 | 1315 |
| RAD51AP1 | 0.725 | 3363 |
| KNTC1 | 0.724 | 2654 |
| PLK1 | 0.724 | 2667 |
| CDC25A | 0.723 | 1546 |
| HMMR | 0.723 | 3992 |
| TROAP | 0.722 | 1606 |
| KIF18B | 0.721 | 2046 |
| DLGAP5 | 0.721 | 3294 |
| NUSAP1 | 0.72 | 2630 |
| KIF2C | 0.72 | 2625 |
| EXO1 | 0.719 | 2004 |
| CENPM | 0.719 | 1978 |
| FOXM1 | 0.717 | 2360 |
| CDC45 | 0.716 | 1931 |
| CDC6 | 0.716 | 2939 |
| CENPF | 0.715 | 1413 |
| CCNA2 | 0.714 | 2717 |
| POLE2 | 0.714 | 2768 |
| MELK | 0.714 | 3062 |
| CDT1 | 0.713 | 2062 |
| KIF4A | 0.713 | 2331 |
| TTK | 0.712 | 3048 |
| SPC25 | 0.711 | 2563 |
| MCM5 | 0.71 | 2878 |
| ZWINT | 0.71 | 2464 |
| PTTG3P | 0.71 | 2225 |
| CDCA8 | 0.71 | 1977 |
| HJURP | 0.709 | 2010 |
| PBK | 0.709 | 4023 |
| ORC1 | 0.709 | 2627 |
| **BRCA1** | **0.708** | **1720** |

^*^Number of times this gene is associated with any gene in the co-expression map; Bold: Genes of interest

**Table S5**

*Co-expressed genes with input gene FANCA and BRCA1*

| **No.** | **Gene Symbol** | **P Value^1^** | **Gene set friends^2^** | **Total friends^3^** | **FANCA^4^** | **BRCA1^4^** |
| --- | --- | --- | --- | --- | --- | --- |
| 1 | FGFR1OP | 3.95E-05 | 2 | 124 | 0.624 | 0.524 |
| 2 | EFCAB11 | 6.50E-05 | 2 | 159 | 0.595 | 0.522 |
| 3 | FDPS | 8.70E-05 | 2 | 184 | 0.596 | 0.525 |
| 4 | CCHCR1 | 1.01E-04 | 2 | 198 | 0.621 | 0.526 |
| 5 | SHMT1 | 1.23E-04 | 2 | 219 | 0.598 | 0.532 |
| 6 | MXD3 | 1.41E-04 | 2 | 234 | 0.621 | 0.523 |
| 7 | INTS7 | 1.52E-04 | 2 | 243 | 0.601 | 0.546 |
| 8 | WDR62 | 1.57E-04 | 2 | 247 | 0.634 | 0.534 |
| 9 | TCOF1 | 2.36E-04 | 2 | 303 | 0.595 | 0.52 |
| 10 | DSCC1 | 2.88E-04 | 2 | 335 | 0.623 | 0.557 |
| 11 | AZI1 | 3.29E-04 | 2 | 358 | 0.623 | 0.535 |
| 12 | CENPI | 3.54E-04 | 2 | 371 | 0.652 | 0.588 |
| 13 | RBL1 | 3.87E-04 | 2 | 388 | 0.635 | 0.553 |
| 14 | CEP152 | 3.95E-04 | 2 | 392 | 0.628 | 0.574 |
| 15 | TMEM106C | 3.97E-04 | 2 | 393 | 0.613 | 0.553 |
| 16 | FANCA | 4.01E-04 | 2 | 395 | 1 | 0.57 |
| 17 | CAD | 4.13E-04 | 2 | 401 | 0.612 | 0.533 |
| 18 | FASN | 4.26E-04 | 2 | 407 | 0.608 | 0.517 |
| 19 | POLE | 4.40E-04 | 2 | 414 | 0.64 | 0.536 |
| 20 | POLA2 | 4.91E-04 | 2 | 437 | 0.614 | 0.595 |
| 21 | DCLRE1B | 5.16E-04 | 2 | 448 | 0.618 | 0.529 |
| 22 | NUP62 | 5.32E-04 | 2 | 455 | 0.596 | 0.524 |
| 23 | HAUS5 | 5.92E-04 | 2 | 480 | 0.612 | 0.525 |
| 24 | H2AFX | 6.12E-04 | 2 | 488 | 0.631 | 0.545 |
| 25 | TOE1 | 6.25E-04 | 2 | 493 | 0.612 | 0.521 |
| 26 | TRAF2 | 7.55E-04 | 2 | 542 | 0.61 | 0.524 |
| 27 | CCNB2 | 8.06E-04 | 2 | 560 | 0.619 | 0.615 |
| 28 | RRP7A | 8.61E-04 | 2 | 579 | 0.594 | 0.519 |
| 29 | CENPJ | 9.69E-04 | 2 | 614 | 0.61 | 0.552 |
| 30 | SLC25A40 | 9.69E-04 | 2 | 614 | 0.603 | 0.535 |
| 31 | LSM4 | 9.75E-04 | 2 | 616 | 0.592 | 0.516 |
| 32 | DTYMK | 9.97E-04 | 2 | 623 | 0.627 | 0.554 |
| 33 | GINS4 | 1.00E-03 | 2 | 625 | 0.646 | 0.602 |
| 34 | C16orf59 | 1.04E-03 | 2 | 637 | 0.635 | 0.581 |
| 35 | RECQL4 | 1.13E-03 | 2 | 663 | 0.688 | 0.6 |
| 36 | PSMC3IP | 1.13E-03 | 2 | 664 | 0.646 | 0.598 |
| 37 | AAAS | 1.14E-03 | 2 | 666 | 0.613 | 0.524 |
| 38 | NCBP1 | 1.17E-03 | 2 | 674 | 0.597 | 0.529 |
| 39 | NOC4L | 1.21E-03 | 2 | 685 | 0.617 | 0.535 |
| 40 | BUB1 | 1.21E-03 | 2 | 687 | 0.676 | 0.621 |
| 41 | RANBP1 | 1.22E-03 | 2 | 688 | 0.629 | 0.548 |
| 42 | APEX2 | 1.22E-03 | 2 | 690 | 0.607 | 0.52 |
| 43 | GTSE1 | 1.23E-03 | 2 | 691 | 0.68 | 0.633 |
| 44 | C17orf53 | 1.23E-03 | 2 | 692 | 0.645 | 0.557 |
| 45 | UCHL5 | 1.25E-03 | 2 | 698 | 0.601 | 0.535 |
| 46 | MPHOSPH9 | 1.30E-03 | 2 | 710 | 0.61 | 0.573 |
| 47 | TIMELESS | 1.30E-03 | 2 | 712 | 0.658 | 0.575 |
| 48 | EXOSC2 | 1.32E-03 | 2 | 716 | 0.637 | 0.543 |
| 49 | SLCO4A1 | 1.32E-03 | 2 | 716 | 0.608 | 0.536 |
| 50 | LRFN4 | 1.33E-03 | 2 | 719 | 0.608 | 0.5 |

^1^Calculated P-value based on "gene set friends", "total number of genes in seed list", and "total friends" using binomial

^2^Number of times this gene is associated with a gene in the seed list

^3^Number of times this gene is associated with any gene in the co-expression map

^4^Co-expression values of each gene in the seed list

**Table S6**

Medical Subject Headings (MeSH) enrichment of 50 genes co-expressed with *FANCA* and *BRCA1*

| **MeSH-Term** | **P-value** | **# Genes (observed)** | **# Genes (expected)** | **# Genes (total)** |
| --- | --- | --- | --- | --- |
| Genomic Instability | 4.42E-04 | 13 | 4.67 | 1820 |
| Microcephaly | 8.33E-04 | 6 | 1.13 | 439 |
| Bloom Syndrome | 3.33E-03 | 3 | 0.30 | 117 |

# Genes (observed) = the number of genes from the input set which have this annotation; # Genes (expected) = the number of genes one would expect to have this annotation based on the input set; # Genes Total = the number of genes from the complete genome which have this annotation.

**Table S7**

Cellular Components (GO) enrichment of 50 genes co-expressed with *FANCA* and *BRCA1*

| **GO-Term** | **GO-Term id** | **P-value** | **# Genes (observed)** | **# Genes (expected)** | **# Genes (total)** |
| --- | --- | --- | --- | --- | --- |
| intracellular organelle part | GO:0044446 | 1.83E-08 | 36 | 17.22 | 6315 |
| organelle part | GO:0044422 | 2.65E-08 | 36 | 17.43 | 6392 |
| centrosome | GO:0005813 | 3.55E-08 | 10 | 0.98 | 360 |

# Genes (observed) = the number of genes from the input set which have this annotation; # Genes (expected) = the number of genes one would expect to have this annotation based on the input set; # Genes Total = the number of genes from the complete genome which have this annotation.

**Legend to Additional Figure**

**Figure S1. Differential cell cycle regulation of FA genes in human EVA-F cells**

(a - left panel) Cells were placed on medium with low serum (0.2% FBS) for 3 days resulting in cell cycle arrest. The addition of high serum medium (10% FBS) released cells resulting in synchronous progression through the cell cycle. Sampled cells at different time points were divided for Fluorescent Activated Cell Sorting (FACS) analysis. Data represents one representative synchronization experiment. Quantitative RT-PCR was performed on RNA samples from different time points and mean fold changes (MFC) were calculated relative to time point zero. Data represents duplo qPCR measurements of one representative synchronization experiment, SEM is indicated. (a - right panel) Cell cycle control *CCNE2* (b) *FANCA* and *FANCG* (c) *FANCB* and *FANCL* (d) *FANCE*, *FANCC*, and *FANCF* (e) *FANCM* (f) *FANCD2* and *FANCI* (g) *BRCA2, PALB2, BRCA1,* and *BRIP1*.
